# “Capgras” Delusions Involving Belongings, Not People, and Evolving Visual Hallucinations Associated with Occipital Lobe Seizures

**DOI:** 10.1155/2018/1459869

**Published:** 2018-03-07

**Authors:** Brandon Lilly, Erika Maynard, Kelly Melvin, Suzanne Holroyd

**Affiliations:** Department of Psychiatry & Behavioral Medicine, Marshall University, Huntington, WV, USA

## Abstract

Capgras syndrome is characterized by the delusional belief that a familiar person has been replaced by a visually similar imposter or replica. Rarely, the delusional focus may be objects rather than people. Numerous etiologies have been described for Capgras to include seizures. Similarly, visual hallucinations, both simple and complex, can occur secondary to seizure activity. We present, to our knowledge, the first reported case of visual hallucinations and Capgras delusions for objects that developed secondary to new onset occipital lobe epilepsy. We then discuss the possible underlying neurologic mechanisms responsible for the symptomatology.

## 1. Introduction

Capgras syndrome is a delusional disorder characterized by the belief that a familiar person has been replaced by a visually similar imposter or replica. Rarely, the delusional focus may be objects rather than people. Associated symptoms can include feelings of depersonalization and derealization. The course of illness may be brief, recurrent, or persistent. The neuropathology of Capgras syndrome is poorly understood but the condition has been linked to a multitude of disorders including brain tumors, neurodegenerative disorders, traumatic brain injuries, vascular disease including hypertensive encephalopathy, infectious diseases, metabolic disorders, endocrinopathies, vitamin deficiencies, drugs and toxins, and epilepsies [1–3]. Various brain regions have been implicated. A functional SPECT brain imaging study of an individual with Capgras syndrome revealed increased perfusion of the left calcarine sulcus of the occipital lobe [4]. Others have reported Capgras associated with temporal lobe dysfunction [5, 6]. Capgras syndrome with epilepsy has been proposed to result either from postictal disinhibition of the dominant hemisphere involved in recognition or from dysfunction of the nondominant hemisphere involved in perceptual integration [1].

Whereas reports of seizures leading to Capgras delusions are rare, other forms of psychosis are more commonly comorbid with epilepsy. Individuals with seizure disorders have a nearly 8-fold increased risk of developing psychosis, with an overall prevalence of about 6% [7]. The most frequently described psychotic symptom associated with epilepsy is visual hallucinations (VH). While the exact causal mechanism is unknown, VH may result from neuroophthalmologic dysfunction due to a variety of etiologies. It has been proposed that VH may be due to direct irritative and inflammatory processes acting on the cortical centers that integrate complex visual information [8].

VH are broadly classified as simple or complex [9]. Simple, also known as elementary, VH consist of “nonformed” objects such as colors, shapes, and geometric designs, whereas complex VH are “formed” objects such as people or animals. Etiologies include ophthalmologic pathologies, neurodegenerative diseases, migraines, substance use, psychiatric illnesses, delirium, and epilepsy [9]. Simple VH have most often been linked with the primary visual cortex in the occipital lobe while complex VH are more often associated with higher order visual association areas in the temporal lobe [10]. For example, for complex partial seizures originating in the temporal lobe, more posterior temporal lobe foci will have more complex VH [11].

Simple VH associated with seizures focused in the occipital lobe are highly stereotypical and usually described as brightly colored circles or spherical patterns that are in motion generally moving across the visual field in a direction contralateral to the seizure focus [9]. Such phenomena are usually binocular and brief. Head and eye deviation, as well as postictal headaches, are common [9].

Here, we describe a patient with evolving visual hallucinations and Capgras delusions for objects that developed secondary to new onset occipital lobe epilepsy.

## 2. Case

A middle-aged female of North East Asian descent was admitted for hypertensive emergency with visual disturbances. She had a BMI of 32. Two days prior to admission, she was evaluated in the emergency department (ED) for blurry vision and found to have elevated blood pressure for which she was given IV metoprolol which successfully lowered her blood pressure. Head CT showed an old stable left thalamic infarct. She was instructed to establish care with a primary care provider to manage her blood pressure. Early on the day of admission, the patient reported waking up and experiencing visual disturbances described as “distorted images” and “blurring of boundaries of common objects” such as a handheld mirror. She reported to a primary care clinic to establish care as previously instructed at the ED. Her blood pressure was found to be 218/105 with complaints of headache and seeing “floating objects” in her visual field. Bedsides, neurological exam did not reveal any focal neurological deficits. She was diagnosed with hypertensive emergency and immediately sent to the local ED. Brain MRI with and without IV contrast confirmed previously noted old left thalamic infarct in the mid-medial thalamus as well as mild punctate high intensity signals in the deep periventricular white matter bilaterally consistent with chronic small vessel ischemic changes. Antihypertensive medications were started to gain control of her blood pressure. Labs revealed mild leukocytosis, hypokalemia, and hyperglycemia. Chest X-ray was significant only for subsegmental atelectasis at the right lung base but was otherwise unremarkable. Urine drug screen was negative. Urinalysis was strongly positive for glucose and ketones with an elevated specific gravity. Vitamin B12, folate, homocysteine, and TSH were within normal limits and lipid profile was unremarkable. CTA Head/Neck was completed and showed a stable 6.3 mm left-sided cavernous carotid aneurysm but was otherwise unremarkable. She was admitted with newly diagnosed Type 2 Diabetes (HgbA1c 14.8).

**Figure 1:**
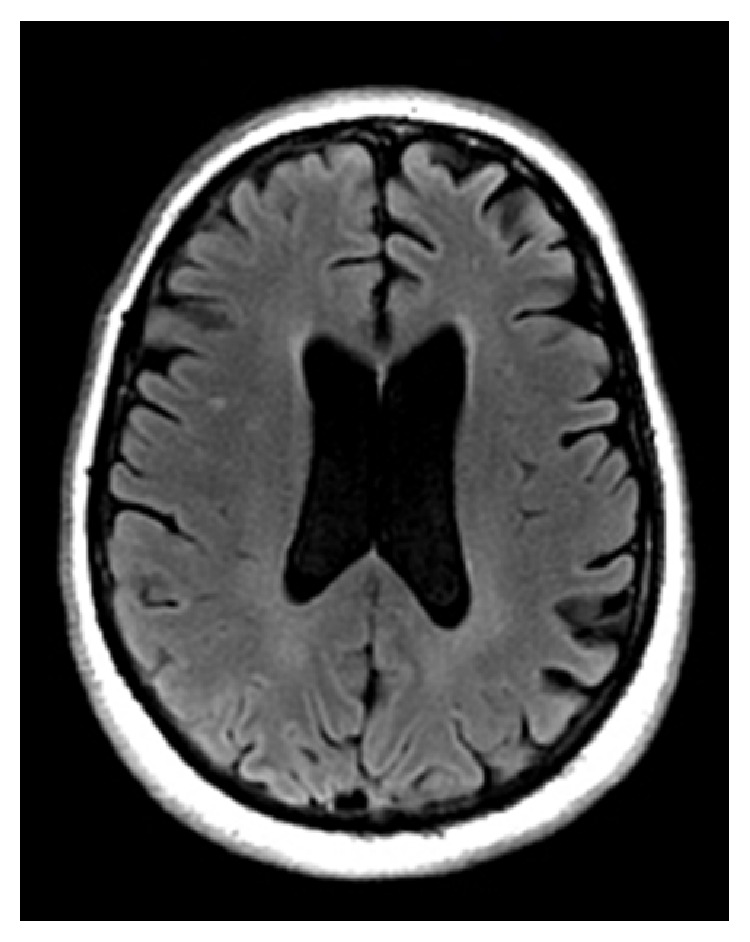
Patient's MRI revealing left thalamic infarct and chronic small vessel ischemic changes.

She then developed left lateral gaze deviation with nystagmus. Repeat head CT showed no apparent acute findings. She described VH of red and green “pinwheels” moving diagonally downward to the left before falling off the visual field. This evolved into complex VH of people and dogs. Neurology was consulted and urgent electroencephalogram (EEG) was completed using the internationally standardized 10–20 system which revealed right occipital lobe seizures starting as fast ictal discharges from the O2 electrode and spreading to T6. Valproic acid and levetiracetam were started. Unfortunately, she developed status epilepticus refractory to lorazepam, requiring intubation and administration of midazolam. After several days, she was extubated and repeat MRI brain (Figure 1) again demonstrated chronic small vessel ischemic changes and the stable old left thalamic infarct. Follow-up EEG did not demonstrate any further epileptic activity and was read as normal.

Interestingly, when presented with her personal belongings after extubation (clothing, shoes, cellphone, and wallet), which had been stored in a hospital provided plastic bag labeled with her name, the patient did not recognize any of them as belonging to her; she adamantly stated she had never seen them before. She did not believe that the cellphone belonged to her despite using the thumbprint recognition feature and viewing text messages which she did remember, nor did she recognize herself in her driver's license photo, even stating that the person pictured was of African descent. For days, she continued to be unable to recognize her belongings as her own, although she could recognize friends and family. The inability to recognize objects as her own was strictly limited to the objects that had been stored in the hospital bag provided at her admission. Following several days of trying to convince her that those were her belongings, including having her call financial institutions to confirm credit card numbers, seeing her address listed on the driver's license, and recognizing text message conversations, the patient reluctantly began to accept her personal belongings, but never felt a true sense that these objects belonged to her. She was discharged on valproic acid 1,000 mg twice daily and levetiracetam 2,000 mg twice daily, as well as newly started metformin, metoprolol tartrate, lisinopril, and amlodipine. She was discharged from the hospital with close follow-up arranged with her primary care provider, neurology, and interventional radiology. The patient reported complete resolution of Capgras-like delusions at neurology follow-up appointments.

## 3. Discussion

Our case describes the onset of visual hallucinations followed by Capgras-like delusions towards personal belongings in the setting of newly diagnosed occipital lobe seizures. To our knowledge, this is the first case to describe the cooccurrence of these symptoms in a patient with occipital lobe epilepsy. Other similar cases reviewed in the literature occurred in the context of primary psychiatric disorder diagnoses, dementia, and other neurodegenerative conditions [12–16].

Anatomically, it is interesting to correlate the patient's symptoms with the location of the epileptic focus and spread. The International 10–20 system for EEG electrode placement was used [17]. Per this standard, O2 corresponds to Brodmann areas 17, 18, and 19 while T6 corresponds to Brodmann areas 19, 27, and 39. Brodmann areas 17, 18, and 19 are located in the occipital lobe and area 17 represents the primary visual cortex. Brodmann area 18 is thought to be primarily a visual association cortex and area 19 includes the lingual gyrus, believed to play an important role in vision processing and dreaming [18]. Per EEG the seizure focus was located in one of these areas and the onset of epileptic activity correlated with her experience of simple VH that progressed to complex. VH were consistent with previously described patterns in that they were stereotypical spheres that moved across the visual field in a direction contralateral to the site of the seizure focus [9]. As the EEG recording is nonspecific in its ability to pinpoint an exact anatomical locus we do not know precisely where her seizure activity originated but it is interesting to note that as the seizure spread her VH became more complex. We speculate that this may have been due to expanding epileptic recruitment of visual association neurons. Some research has demonstrated an association between complex VH and areas 18 and 19 [10, 11].

We do know that the direction of spread was from O2 to T6 and given that both EEG placements overlap area 19 we can conclude that neuronal excitation spreads in a rostral fashion. T6 further overlays areas 27 and 39. Area 27 encompasses a portion of the parahippocampal gyrus, important for memory encoding and retrieval. Area 39 is parietal rather than occipital and includes the angular gyrus, which is involved with language, attention, and memory retrieval and is implicated in out of body experiences.

Our patient first voiced Capgras delusions following extubation and cessation of seizure activity. At that point, she no longer experienced VH. We speculate that our patient sustained an insult, perhaps ischemic or possibly an epileptic-related insult, which affected a brain area that regulates visual-emotional recognition or the affective sense of personal belonging and ownership. Given that the areas involved include primary vision processing, visual association, and encoding and retrieval of visual information it is likely that neural connectivity within one or more of these locations became disrupted.

Consideration of the Capgras delusional process should take into account visual information processing and association at multiple levels. Individuals with Capgras are able to input visual data and associate that data with prior memory associations (i.e., “I see a thing, that thing is a cell phone, that cell phone looks like mine”). However, a deeper level of association appears to be disrupted which is the affective sense of personal belonging (i.e., “That cell phone feels like mine”). For our patient, the involvement of areas 19, 27, and 39 is intriguing given their roles in vision processing, memory encoding and retrieval, and their connectivity with the limbic system and thus affective content. Area 39's involvement is particularly compelling given that some patients with Capgras report depersonalization or derealization.

The disruption of affective association as described above would align with theories of delusion formation in general, as described by Coltheart. That is, the two factors are involved in the formation and maintenance of delusions: Factor 1, which is responsible for the delusional belief's content (i.e., these things are not mine), and Factor 2, which is responsible for the persistence of said delusion despite evidence to the contrary (i.e., patient's use of thumbprint recognition to unlock their cell phone, recognition of text conversations in their cell phone). In this case, Factor 1 was the initial insult resulting in disruption of neural connectivity within visual association areas. Factor 2 could potentially be explained by executive dysfunction caused by preexisting small vessel ischemic disease noted in the frontal lobe. Several cases of Capgras have been described which feature frontal lobe abnormalities [19–23].

It is possible that other brain regions were responsible or contributed to our patient's condition. An old left thalamic infarct in the medial thalamus was seen on MRI. While most cases of Capgras are associated with either right or bilateral hemispheric brain abnormalities, there have been reports in the literature of Capgras associated with left hemispheric abnormalities and the laterality of Capgras etiology in the brain is actively debated [22–25]. Connections between the prefrontal cortex (PFC) and medial thalamus were also considered given the PFC's role in executive functioning, attention, and memory. However, some evidence suggests that these circuits are more concerned with the processing of new rather than existing encoded information [26]. Lastly, some association between the medial thalamus and familiarity-driven recognition is noted in the literature. However, this is still in debate and recent work casts doubt on this association, calling for reappraisal of models of said association [26, 27].

It is worth noting that we considered other causes for our patient's symptoms to include delirium and posterior reversible encephalopathy syndrome (PRES). Our patient did not present with the waxing and waning of consciousness and cognition characteristic of delirium. Further, findings on MRI did not support a diagnosis of PRES.

Our case highlights a patient who experienced both visual hallucinations and Capgras delusions after new onset of occipital lobe epilepsy. To our knowledge, this is the first report to describe the occurrence of these phenomena in tandem following occipital lobe seizures. Though the exact causative mechanism underlying these conditions remains unknown, it is hoped that this report will add something substantive to the existing literature.

## References

[1] Joshi D., Koirala S., Lamichhane S., Paladugu A., Johal R., Lippmann S. (2010). Capgras syndrome in postictal delirium. *Psychiatry (Edgmont)*.

[2] Sadock B., Sadock V., Ruiz P. (2015). Delusional disorder and shared psychotic disorder. *Synopsis of Psychiatry*.

[3] Alexander M. P., Stuss D. T., Benson D. F. (1979). Capgras syndrome: a reduplicative phenomenon. *Neurology*.

[4] Mackie J., Ebmeier K. P., O’Carroll R. E. (1994). An MRI, SPECT and neuropsychological study of a patient presenting with capgras syndrome. *Behavioural Neurology*.

[5] Ardila A., Rosseli M. (1988). Temporal lobe involvement in capgras syndrome. *International Journal of Neuroscience*.

[6] Josephs K. A. (2007). Capgras syndrome and its relationship to neurodegenerative disease. *JAMA Neurology*.

[7] Pelak V. S. Approach to the patient with visual hallucinations, 2016. https://www.uptodate.com/contents/approach-to-the-patient-with-visual-hallucinations?source=search_result&search=visual%20hallucinations&selectedTitle=1~113.

[8] Manford M., Andermann F. (1998). Complex visual hallucinations. Clinical and neurobiological insights. *Brain*.

[9] Hilger E., Zimprich F., Pataraia E. (2016). Psychoses in epilepsy: A comparison of postictal and interictal psychoses. *Epilepsy & Behavior*.

[10] Foerster L. (1931). The cerebral cortex in man. *Lancet*.

[11] Slade P. (1976). Editorial: Hallucinations.. *Psychological Medicine*.

[12] Islam L., Piacentini S., Soliveri P., Scarone S., Gambini O. (2015). Capgras delusion for animals and inanimate objects in Parkinson's Disease: A case report. *BMC Psychiatry*.

[13] Bhide A. V. (1994). A capgras like state for inanimate objects: two case reports. *Indian Journal of Psychiatry*.

[14] Nejad A. G., Toofani K. (2006). A variant of Capgras syndrome with delusional conviction of inanimate doubles in a patient with grandmal epilepsy. *Acta Neuropsychiatrica*.

[15] Rastogi S. C. (1990). A variant of Capgras syndrome with substitution of inanimate objects. *The British Journal of Psychiatry*.

[16] Abed R. T., Fewtrell W. D. (1990). Delusional misidentification of familiar inanimate objects. A rare variant of capgras syndrome. *The British Journal of Psychiatry*.

[17] Malmivuo J., Plonsey R. (1995). *Bioelectromagnetism: Principles and Applications of Bioelectric and Biomagnetic Fields*.

[18] Teeple R. C., Caplan J. P., Stern T. A. (2009). Visual hallucinations: differential diagnosis and treatment. *Primary Care Companion to the Journal of Clinical Psychiatry*.

[19] Coltheart M. (2010). The neuropsychology of delusions. *Annals of the New York Academy of Sciences*.

[20] Luca M., Bordone A., Luca A., Patti A., Sortino G., Calandra C. (2013). Clinical features and imaging findings in a case of Capgras syndrome. *Neuropsychiatric Disease and Treatment*.

[21] Bobes M. A., Góngora D., Valdes A. (2016). Testing the connections within face processing circuitry in Capgras delusion with diffusion imaging tractography. *NeuroImage: Clinical*.

[22] Huang T.-L., Liu C.-Y., Yang Y.-Y. (1999). Capgras syndrome: Analysis of nine cases. *Psychiatry and Clinical Neurosciences*.

[23] Pandis C. C., Poole N. (2017). 15Capgras delusion: a meta-analysis of case reports in the english language. *Journal of Neurology, Neurosurgery & Psychiatry*.

[24] Fils J. M., Stewart J. T. (2011). Capgras syndrome related to left-hemisphere injury. *The Journal of Neuropsychiatry and Clinical Neurosciences*.

[25] Peña-Salazar C., Cendrós P., Escoté S. (2014). Capgras syndrome with left hemisphere neurological damage. *The Journal of Neuropsychiatry and Clinical Neurosciences*.

[26] Mitchell A. S., Chakraborty S. (2013). What does the mediodorsal thalamus do?. *Frontiers in Systems Neuroscience*.

[27] Danet L., Pariente J., Eustache P. (2017). Medial thalamic stroke and its impact on familiarity and recollection. *eLife*.

